# Chronic Postoperative Endophthalmitis: A Review of Clinical Characteristics, Microbiology, Treatment Strategies, and Outcomes

**DOI:** 10.1155/2012/313248

**Published:** 2012-02-22

**Authors:** Fadi Maalouf, Marwan Abdulaal, Rola N. Hamam

**Affiliations:** Ocular Immunology and Uveitis Service, Department of Ophthalmology, American University of Beirut, Riad El Solh 11072020, Beirut, Lebanon

## Abstract

Chronic postoperative endophthalmitis (CPE) is a delayed infectious intraocular inflammation process that occurs more than six weeks after ocular surgery and frequently masquerades as autoimmune uveitis. These cases are at risk of delayed diagnosis and erroneous long-term treatment with corticosteroids. This paper aims to review the epidemiology, microbiology, clinical characteristics, diagnosis, management strategies, and outcome of chronic postoperative endophthalmitis. The incidence of CPE is still uncommon, and multiple pathogens have been reported with varying frequencies. Review of the literature reveals that CPE cases have a high incidence of visual impairment and recurrence rate might be decreased with aggressive surgical approach.

## 1. Introduction and Definitions

Endophthalmitis is an uncommon but sight-threatening intraocular inflammation that may be due to a noninfectious process or may be caused by an infectious organism. It is a term used to describe intraocular inflammation that involves the vitreous cavity and the anterior chamber of the eye and can involve other adjacent ocular tissues such as the choroid or retina, sclera or cornea [1]. In infectious endophthalmitis, the organism might reach the eye from other infected sites in the body through hematologic seeding and in these cases it is labeled endogenous endophthalmitis. More commonly, the organism is exogenous and gains access to the intraocular environment [2]. According to the Endophthalmitis Vitrectomy Study, postoperative endophthalmitis is divided generally into two types: acute and chronic. Acute postoperative endophthalmitis is defined as infections within 6 weeks of surgery; on the other hand, chronic postoperative endophthalmitis is defined as infections after 6 weeks of surgery [3].

The term chronic postoperative endophthalmitis (CPE) was first coined in 1986 in a case series of 15 patients by Meisler et al. [4]. The inflammation is usually indolent and may persist for months. It is often misdiagnosed as noninfectious iritis where it improves initially with topical corticosteroid therapy while flaring whenever corticosteroids are tapered or stopped [5]. This is in contrast to acute postoperative endophthalmitis, which presents as a single episode of severe inflammation with an acute onset that usually follows surgery by a few days but can be delayed more than a week in some cases. As such, acute and chronic postoperative endophthalmitis are two clearly different clinical entities [2, 4, 5].

## 2. Epidemiology

Postoperative endophthalmitis is an uncommon complication of any ocular surgery. The reported incidence of postoperative endophthalmitis ranges from 0.01% to 0.367%, with incidence varying among different surgical procedures and across studies and different countries [1, 6–11]. Most of postoperative endophthalmitis studies were conducted on cases after cataract surgery, being the most commonly performed surgery in ophthalmology [11]. In a large meta-analysis, 3 140 650 cataract extraction cases were reviewed for the incidence of endophthalmitis after cataract surgery worldwide in the period between 1964 and 2003 [12]. The analysis showed an increase in the incidence of postsurgical endophthalmitis from 0.087% in the 1990s to 0.265% in the 2000s, and this was attributed to the change in surgical technique towards clear corneal sutureless wounds that allow exogenous organisms easy access to the intraocular space.

Furthermore, postoperative endophthalmitis has been reported after pars plana vitrectomy, penetrating keratoplasty, trabeculectomy, and glaucoma drainage device surgeries. Endophthalmitis also has been reported following external ocular surgeries such as scleral buckle, pterygium excision, and strabismus surgeries [11]. The highest endophthalmitis rate was found in surgical procedures associated with cataract extraction reaching 0.367%; on the other hand, pars plana vitrectomy was found to have the lowest incidence rate with only 0.04% especially after using microincision technique [11, 13].

The data regarding the incidence of chronic postoperative endophthalmitis are still lacking. But this form of postoperative endophthalmitis appears less common than the acute variety [14]. Some reports estimated the ratio of acute to chronic postoperative endophthalmitis to be between 5 : 1 and 2 : 1, indicating that the incidence rate of chronic postoperative endophthalmitis can be 5 per 10000 [15]. In one single-center study, the reported rate of chronic onset endophthalmitis following cataract surgery was 0.017% [16].

## 3. Etiology, Microbiology, and Pathogenesis

The organisms causing chronic postoperative endophthalmitis tend to be different from the acute form pathogens [14]. They are usually indolent bacteria or fungus with low virulence. CPE was originally considered to be a reaction to the remaining native lens tissue and was consequently called toxic lens syndrome or phacoanaphylactic endophthalmitis [17]. However, studies of removed lens capsules revealed small gram-positive rods, consistent with *Propionibacterium acnes*, adherent to the capsular remnants [17].

A variety of organisms have been implicated in chronic postoperative endophthalmitis (Table 1), with *Propionibacterium* species accounting for the majority of cases (41 to 63%) followed by coagulase-negative *Staphylococcus* and fungus [5, 16, 18].

*Propionibacterium acnes*, formerly known as *Corynebacterium parvum*, is a variably staining, gram-positive, pleomorphic, and anaerobic bacillus. As its name suggests, *P. acnes* is associated with chronic skin infections and with the contamination of a variety of prosthetic devices [19, 20]. Despite being a potent stimulant of the immune system, *P. acnes* is largely resistant to the killing mechanisms of monocytes and neutrophils, which enables it to persist intra-cellularly after phagocytosis [21].

Reviewing the largest three case series of CPE revealed that 48% of the cases are caused by *P. acnes*, followed by fungal organisms in 21% of the cases and gram-positive species in 16% of the cases (Table 2).

Some case reports have also isolated *Actinomyces*, *Nocardia*, *Achromobacter*, *Cephalosporium*, *Acremonium*, *Paecilomyces*, *Ochrobactrum* and *Aspergillus* species as causes of CPE [22, 29–31]. In some of these organisms such as *Staphylococcus epidermidis,* and *Propionibacterium acnes*, the clinical course of the disease may be affected by factors such as host characteristics or inoculum size [2, 5].

Routes of bacterial entry are believed to include intraoperative irrigation fluids, surgical instruments, and inadvertently placing the intra-ocular lens on external ocular surfaces [32, 33]. The anterior chamber possesses an efficient mechanism of clearing small bacterial loads, so the currently unexplainable failure of this mechanism may be one of a multitude of unknown factors in postoperative bacterial endophthalmitis [32, 34]. Known risk factors include vitreous communication (e.g., through a posterior capsular tear or YAG capsulotomy), certain IOL prosthetics, and diabetes [35–38].

Fungal endophthalmitis is uncommon in the postoperative setting, with most of the cases being attributable to *Candida* species [5]. As such, most fungal endophthalmitis cases are the result of infection by filamentous fungi, and a minority is the result of molds [39]. Fungi possess resistant cell walls that enable them to flourish in the eye indefinitely shielded from immune attack and antibiotic therapy making the management of these cases particularly challenging [40, 41].

## 4. Symptoms and Clinical Finding

The clinical picture of CPE is that of a recurrent and often low-grade uveitis occurring months or even years after the inciting surgical event. Uveitis typically starts two to three months postoperatively and involves the anterior chamber initially with progression to the vitreous as the disease advances. Pain or discomfort may or may not be present in CPE, while decreased vision is found in nearly all patients. Inflammation is usually steroid responsive initially but recurs after medication tapering, while it paradoxically worsens with steroids in the case of some fungal infections [42]. The clinical course in CPE is similar to that of phaeoantigenic uveitis and has been suggested to be a result of an immune reaction to the presence of both residual lens material and bacteria [43, 44]. A slit lamp eye examination will reveal white blood cells in the anterior chamber. The uveitis may be granulomatous with large precipitates on the cornea or intraocular lens and often without a frank hypopyon, but a microhypopyon may be visible by gonioscopy. A white intracapsular plaque representing retained lens particles and sequestered organisms is highly suspicious of an infectious process [14]. The plaque is commonly observed especially in association with *Propionibacterium* species and less frequently with other bacterial or fungal infections [16, 29, 45, 46]. Vitreous activity is usually mild but can be dense and diffuse particularly with *Staphylococcus epidermidis *[5]. CPE of fungal etiology is usually characterized by “pearls-on-a-string” or “fluff balls” near the capsular remnant and also with stringy white infiltrates although both are not pathognomonic [5, 14].

## 5. Diagnostic Approach

The diagnosis of CPE is challenging given the difficulties faced in isolating the causative organism. It is based on clinical suspicion supported by cultures of the aqueous or posterior lens capsule or vitreous biopsy [47]. When CPE is suspected, aqueous and/or vitreous samples should be obtained for analysis. The sampling could be performed using needle aspiration of 0.01 mL of the aqueous fluid or 0.02 mL of the vitreous. In case the vitreous needle aspiration was not successful (dry tap), mechanical biopsy of the vitreous through a pars plana vitrectomy could be performed. The obtained sample should be analyzed with gram stain, culture, and identification of antimicrobial sensitivities [14]. The appropriate anaerobic medium should be used when necessary and Giemsa and fungal cultures should be obtained in case a fungus is suspected. The highest diagnostic yield is achieved by sampling the white plaque in the posterior lens capsule if present, utilizing a special culture medium, as well as prolonging the culture time to several weeks to cover the slow-growing organisms implicated in CPE [14, 48]. In culture negative cases, the additional use of polymerase chain reaction was reported to aid in the identification of the organism [49]. The utilization of a universal bacterial primer could be of help in this setting.

CPE differential diagnosis spectrum includes noninfectious causes such as lens-induced uveitis secondary to retained cortical material, IOL-induced uveitis secondary to implant malposition causing iris chafing and chronic inflammation, and sympathetic ophthalmia or other causes of uveitis unrelated to surgery [50, 51].

## 6. Treatment Strategies and Outcomes

The indolent nature of the organisms and their sequestration within the capsule protected from host defenses along with their different virulence factors make it hard to define a treatment protocol for CPE or extrapolate the guidelines set for acute postoperative endophthalmitis [14].

Different modalities of treatment approaches have been reported, and they range from (1) intraocular antibiotics injection (IOAB) only to, (2) pars plana vitrectomy (PPV) with IOAB to, (3) PPV with IOAB and partial capsulectomy to, (4) PPV with IOAB and total capsulectomy with IOL removal or exchange [5, 16, 18, 28]. In addition, some advocate waiting for culture, gram stain, and sensitivity data to allow for directed therapy in cases where the inflammation is not considered severe [2]. 

Two intraocular antibiotics injection approaches have been described either into the capsular bag or simultaneously into the aqueous and the vitreous [52, 53].

Some reports suggest tailoring treatment options to the severity of presenting signs and symptoms where mild cases are to be managed with intraocular cultures followed by intravitreal antibiotics while using repeated intraocular antibiotic and pars plana vitrectomy with partial capsulectomy in recurrent cases [42]. Another approach depends on the type of the isolated organism whereby *S. epidermidis* could be treated with intraocular antibiotic injections alone while *P. acnes* would require surgical intervention with pars plana vitrectomy, capsulectomy and possible removal or exchange of the IOL in addition to intraocular antibiotic injection [13, 20, 44]. This is based on the fact that multiple reports described high rate of recurrence when *P. acnes* CPE was treated with intravitreal antibiotics alone [20, 44].

Since at the time of the initial antibiotic injection the organism is usually unknown, the initial approach to consider in the empiric treatment of chronic postoperative endophthalmitis, when fungal infection is not suspected, is intravitreal vancomycin (1 mg/0.1 mL) owing to its broad coverage of gram-positive bacteria and methicillin-resistant *Staphylococci*. *P. acnes*, the most commonly described causative organism of CPE, is also sensitive to vancomycin but not to aminoglycosides [14, 15]. It has also been reported to have good susceptibility to carbapenems (meropenem and ertapenem) in vitro [54]. Accordingly, the treatment should be modified as sensitivity studies become available [15].On the other hand, the benefit of systemic and topical antibiotic use remains controversial in CPE [14].

A cross-sectional review of four of the biggest case series on delayed-onset endophthalmitis revealed differences in outcomes that can be attributed to causative organism, initial treatment modality, as well as the extent of intervention [5, 16, 18, 28]. A total of 98 patients with CPE were reported in these series. The overall visual outcome is calculated to be 20/40 or better in about 46% of the cases while 54% ended up with varying degrees of visual impairment, all irrespective of the stratifying factors mentioned above (Table 3).

All four case series indicate that an infection with *P. acnes *or gram-positive organisms was associated with a better visual outcome (better than 20/40 in 54.5% and 50% of the overall cases, resp.) (Table 4). Fungal infection was associated with a more unfavorable prognosis where visual impairment was precipitated in more than 60%, and more than 20% had severe visual impairment (worse than 5/200). In the same pool of patients, the recurrence rate differed in relation to the initial treatment modality (Table 5). The highest recurrence was seen in cases where the initial treatment consisted of antibiotics alone (90%). Starting therapy with pars plana vitrectomy and antibiotics decreased the recurrence in all series, while adding posterior capsulectomy to pars plana vitrectomy and antibiotics as an initial management further decreased the recurrence rate to 42%. As a trend, all case series showed that recurrence rate decreased uniformly in correlation with a more aggressive management strategy (Table 6), whereby the overall calculated recurrence rate, when combined PPV, IOAB, total capsulectomy, and removal or exchange of the IOL was performed at any time during followup, decreased to as low as 5% compared to 68% recurrence rate when PPV was combined with IOAB alone (Table 6).

Chronic fungal postoperative endophthalmitis carries a poor prognosis and there is no standard management available for treating this very rare condition. Current approach includes pars plana vitrectomy, intravitreal amphotericin (5–10 mg/0.1 mL) or voriconazole, and a systemic antifungal drug [55–57]. The indolent course of the chronic fungal postoperative endophthalmitis might benefit from prolonged systemic treatment with an antifungal (6 weeks–6 months) [57]. Topical antifungal agents (natamycin 5%) are started when required, especially in cases of corneal involvement [57].

In conclusion, chronic postoperative endophthalmitis should always be in the differential of recurrent inflammation in a previously operated eye. A worsening course of inflammation despite treatment is particularly alarming. Effort should be directed towards finding a definitive diagnosis in this setting through obtaining intraocular samples for analysis early enough to institute aggressive treatment and avoid recurrence and poor outcome.

## Figures and Tables

**Table 1:** Infectious pathogens isolated in chronic postoperative endophthalmitis [5, 16, 22–27].

Bacterial pathogens	
*Propionibacterium acn*es	*Ochrobactrum anthropi*
*Staphylococcus *species	*Hafnia alvei*
*Corynebacterium*	*Sphingomona spaucimobilis*
*Nocardia*	*Mycobacterium chelonae*
*Cephalosporium and Acremonium*	*Pseudomonas stutzeri*
*Paecilomyces*	*Achromobacter*

Fungul pathogens	

*Aspergillus* species	*Fonsecaea pedrosoi*
*Candida *species	*Paecilomyces* species
*Curvularia lunata*	*Acremonium strictum*

**Table 2:** Percentage of organisms reported in different case series of chronic postoperative endophthalmitis [5, 18, 28].

Pathogens	Shirodkar	Al-Mezaine	Fox	Percentage
*Propionibacterium acne*	11	7	12	48.3%
*Gram-positive species*	3	3	4	16%
*Gram-negative species*	3	1	0	6.4%
*Mycobacteria*	2	0	0	3.2%
*Fungal species*	7	3	3	21.3%
*Mixed*	0	3	0	4.8%

**Table 3:** Visual acuity outcomes reported in four major series of chronic postoperative endophthalmitis [5, 16, 18, 28].

VA outcome	Fox (*n* = 19)	Clark (*n* = 36)	Al-Mezaine (*n* = 17)	Shirodkar (*n* = 26)	Overall (*n* = 98)
≥20/40	9 (47.3%)	18 (50%)	5 (29.4%)	13 (50%)	45 (45.9%)
20/50 ≥ 20/400	6 (31.5%)	10 (28%)	4 (23.5%)	6 (23%)	26 (26.5%)
<20/400 ≥ 5/200	1 (5.2%)	2 (5%)	2 (11.7%)	2 (7.7%)	7 (7.1%)
<5/200-NLP	3 (15.8%)	6 (17%)	6 (35%)	5 (19.2%)	20 (20.4%)

**Table 4:** Visual acuity outcomes by causative organism in chronic post operative endophthalmitis [5, 16, 18, 28].

Organism	≥20/40	20/50 ≥ 20/400	<20/400 ≥ 5/200	<5/200-NLP
*P. acnes* (*n* = 66)	36/66 (54.5%)	20/66 (30%)	2 (3%)	8/66 (12%)
Gram positive (*n* = 10)	5/10 (50%)	2/10 (20%)	1/10 (10%)	1/10 (10%)
Fungal (*n* = 13)	5/13 (38.5%)	3/13 (23%)	2/13 (15%)	3/13 (23%)
*Others (*n* = 9)	1/9 (11%)	2/9 (22%)	0	6/9 (66.6%)

*Others: gram-negative, mycoplasma, and mixed organisms.

**Table 5:** Recurrence rate of chronic post operative endophthalmitis with different initial treatment modalities [5, 18, 28].

Initial treatment	Fox (*n* = 19)	Clark (*n* = 36)	Shirodkar (*n* = 26)	*Overall (*n* = 62)
IOAB only	4/5 (80%)	12/12 (100%)	2/3 (66%)	18/20 (90%)
PPV + IOAB	2/2 (100)	5/10 (50%)	8/10 (80%)	15/22 (68%)
PPV + PC+ IOAB	5/11 (45%)	2/14 (5.5%)	9/13 (69%)	16/38 (42%)
PPV + IOL exchange	0/1 (0%)	None	None	0/1 (0%)

*AL-Mezaine review series was not included since it did not mention the recurrence rate after initial treatment.

**Table 6:** Recurrence rate of chronic post operative endophthalmitis with different surgical interventions [5, 16, 18, 28].

Treatment modality*	Fox (*n* = 19)	Clark (*n* = 36)	Al-Mezaine (*n* = 17)	Shirodkar (*n* = 26)	Overall (*n* = 98)
PPV + IOAB	5/7 (71%)	5/10 (50%)	1/3 (33.3%)	10/12 (83%)	15/22 (68%)
PPV + PC + IOAB	1/9 (11%)	4/21 (19%)	0	9/13 (69%)	14/43 (32%)
PPV + TC + IOL exchange	0/4 (0%)	0/7 (0%)	0/4 (0%)	1/7 (14%)	1/22 (4.5%)
PPV + TC + no IOL	0	0/5 (0%)	0/1 (0%)	1/12 (8%)	1/18 (5.5%)

*At any time of treatment (initial, secondary, or tertiary intervention).
